# Disubstituted piperazine analogues of trifluoromethylphenylpiperazine and methylenedioxybenzylpiperazine: analytical differentiation and serotonin receptor binding studies

**DOI:** 10.1080/20961790.2018.1445497

**Published:** 2018-04-05

**Authors:** Jack DeRuiter, Ashleigh Van Cleave, Audinei de Sousa Moura, Younis Abiedalla, C. Randall Clark

**Affiliations:** a Department of Drug Discovery and Development, Harrison School of Pharmacy, Auburn University, Auburn, AL, USA; b Department of Pharmacy, Federal University of Piaui, Teresina, Brazil; c Faculty of Pharmacy, Department of Medicinal Chemistry, Omar Al-Mukhtar University, El-Beida, Libya

**Keywords:** Forensic science, forensic toxicology, gas chromatography–mass spectrometry, gas chromatography–infrared spectroscopy, regioisomers, receptors, serotonin, disubstituted piperazines

## Abstract

A series of N,N-disubstituted piperazines were synthesized containing the structural elements of both methylenedioxybenzylpiperazine (MDBP) and trifluoromethylphenylpiperazine (TFMPP) in a single molecule. These six potential designer drug molecules having a regioisomeric relationship were compared in gas chromatography-mass spectrometry (GC–MS), gas chromatography-infrared spectroscopy and serotonin receptor affinity studies. These compounds were separated by capillary gas chromatography on an Rxi®-17Sil MS stationary phase film and the elution order appears to be determined by the position of aromatic ring substitution.

The majority of electron ionization mass spectral fragment ions occur via processes initiated by one of the two nitrogen atoms of the piperazine ring. The major electron ionization mass spectrometry (EI-MS) fragment ions observed in all six of these regioisomeric substances occur at *m*/*z* = 364, 229, 163 and 135. The relative intensity of the various fragment ions is also equivalent in each of the six EI-MS spectra. The vapour phase infrared spectra provide a number of absorption bands to differentiate among the six individual compounds on this regioisomeric set. Thus, the mass spectra place these compounds into a single group and the vapour phase infrared spectra differentiate among the six regioisomeric possibilities.

All of the TFMPP–MDBP regioisomers displayed significant binding to 5-HT_2B_ receptors and in contrast to 3-TFMPP, most of these TFMPP–MDBP isomers did not show significant binding at 5-HT_1_ receptor subtypes. Only the 3-TFMPP-3,4-MDBP (Compound 5) isomer displayed affinity comparable to 3-TFMPP at 5-HT_1A_ receptors (*K_i_* = 188 nmol/L).

## Introduction

The compounds in this paper are hybrid analogues containing the structural fragments of both 1-(3-trifluoromethylphenyl) piperazine (3-TFMPP) and 1-(3,4-methylenedioxybenzyl) piperazine (3,4-MDBP) drugs of abuse in a single molecule. Benzylpiperazine (BZP) and 3-TFMPP are among the most common piperazines encountered in clandestine drug samples [1,2]. The combination of these two drugs has been described as producing effects similar to 3,4-methylenedioxymethamphetamine (MDMA) [3]. The 3-TFMPP isomer is reported to interact with serotonergic receptors and the affinity and selectivity at these receptors are dependent on the nature and position of the aromatic substituents [2].

Legal control has stimulated the development of numerous designer drug analogues and these molecular modifications are often an attempt to obtain novel psychoactive substances not specifically controlled by existing regulations. This phenomenon has been observed in synthetic cannabinoids [4,5], phenethylamine-type drugs of abuse including amphetamine [6], MDMA [7,8], cathinone derivatives or bath salts [9,10] and other clandestine drug series [11,12]. These designer-style structural alterations have been encountered in the piperazine compounds, and the modification of the controlled substance N-BZP has led to 1-(3,4-MDBP), the methylenedioxy analogue of BZP.

The ability to distinguish between regioisomers is extremely important in forensic drug identification of synthetic designer drugs. Recently, our group has published reports describing the differentiation of 3-TFMPP from the regioisomeric 2- and 4-TFMPPs by gas chromatography with infrared (IR) detection and gas chromatography–mass spectrometry (GC–MS) [13,14]. In some cases, the regioisomerism is within that portion of the molecule yielding the major electron ionization mass spectrometry (EI-MS) fragment ions thus leading to spectral equivalency. Furthermore, these closely related substances often show similar chromatographic elution properties, even co-elution under some experimental conditions. Those substances co-eluting in the chromatographic system and having common mass spectral fragment ions could be misidentified.

The N, N-disubstitution of the two secondary amine nitrogens of piperazine with the methylenedioxybenzyl and trifluoromethylphenyl groups yields molecules with the structural elements of MDBP and TFMPP in a single molecule. A recent study [15] compared the cardiotoxic effects of a series of monosubstituted piperazines including 3-TFMPP and 3,4-MDBP. The cytotoxicity of 3-TFMPP was more than 10-fold greater than that of 3,4-MDBP. Both drugs caused a significant increase in intracellular calcium levels and a decrease in mitochondrial membrane potential.

**Figure 1.**
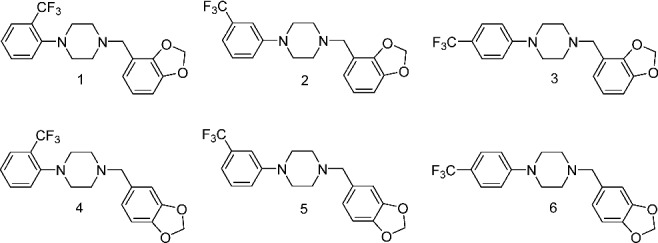
Structures of the N,N-disubstituted piperazine derivatives, N-2,3-methylenedioxy-benzyl-2-, 3- and 4-trifluoromethylphenylpiperazines (Compounds 1–3) and N-3,4-methylene-dioxybenzyl-2-, 3- and 4-trifluoromethylphenylpiperazines (Compounds 4–6).

Differentiation of the regioisomeric N,N-disubstituted piperazines (Compounds 1–6 in Figure 1) via common forensic analytical techniques is the focus of this study. These compounds represent possible designer analogues in this series and the synthetic precursors for the preparation of these six substances are commercially available. The aim of this study is to establish analytical methods for the identification of each of these regioisomeric compounds and compare the serotonin receptor affinities for these potential designer substances.

## Experimental

### Instrumentation

The GC–MS system consisted of an Agilent Technologies (Santa Clara, CA) 7890A gas chromatograph and an Agilent 7683B auto-injector coupled with a 5975C VL Agilent mass selective detector. The mass spectral scan-rate was 2.86 scans/s. The GC was operated in splitless injection mode with a helium (ultra-high purity, grade 5, 99.999%) flow-rate of 0.7 mL/min and the column-head pressure was 10 psi. The MS was operated in the electron ionization (EI) mode using an ionization voltage of 70 eV and a source temperature of 230 °C. The GC injector was maintained at 300 °C and the transfer line at 325 °C. Chromatographic separation was carried out on a column (30 m × 0.25 mm i.d.) coated with a midpolarity Crossbond® silarylene phase similar to 50% phenyl, 50% dimethyl polysiloxane (Rxi®−17Sil MS; *d_f_* = 0.25 *μ*m) purchased from Restek Corporation (Bellefonte, PA). Samples were dissolved and diluted in high-performance liquid chromatography-grade acetonitrile (Fisher Scientific, Fairlawn, NJ) and introduced via the auto-injector using an injection volume of 1 *μ*L. The EI-MS spectra and chromatographic separation of the six regioisomeric compounds were obtained using a temperature programme consisting of an initial hold at 80  °C for 1.0 min, ramped up to 300 °C at a rate of 30 °C/min and held at 300 °C for 0.5 min, then ramped up to 340 °C at a rate of 5 °C/min and held at 340 °C for 5.0 min.

Gas chromatography–IR spectroscopy (GC–IR) studies were carried out on a Hewlett-Packard 6890 Series gas chromatograph and a Hewlett-Packard 7683 series auto-injector coupled with an infrared detector (IRD) Model IRD-3 detector obtained from Analytical Solutions and Providers (ASAP, Covington, KY). The vapour phase IRD spectra were recorded in the range of 4 000–550 cm^−^^1^ with a resolution of 8 cm^−^^1^ and a scan-rate of 1.5 scans/s. The IRD flow cell and transfer line temperatures were maintained at 280 °C and the GC was operated in the splitless injection mode with a carrier gas helium (ultra-high purity, grade 5, 99.999%) flow-rate of 0.7 mL/min. The column used was a 30 m × 0.25 mm i.d. capillary coated with 0.10 *µ*m Crossbond®, selectivity close to 5% diphenyl, 95% dimethyl polysiloxane (Rxi®−5Sil MS) purchased from Restek Corporation (Bellefonte, PA). The temperature programme consisted of an initial temperature of 100 °C for 1 min, ramped up to 230 °C at a rate of 20 °C/min followed by a hold at 230 °C for 15 min.

### Synthetic methods

All laboratory reagents and chemicals were obtained from Aldrich Chemical Co. (Milwaukee, WI) or Fisher Scientific (Atlanta, GA). Samples of 2-, 3- and 4-TFMPPs were purchased from commercial sources. The general procedure for the synthesis of these regioisomeric N,N-disubstituted piperazines begins with the appropriate TFMPP and the commercially available substituted benzaldehydes (2,3- and 3,4-methylenedioxybenzaldehydes) as starting materials. The desired N,N-disubstituted piperazine regioisomers were synthesized by stirring a solution of the appropriate aldehyde in methanol with the appropriate TFMPP and sodium triacetoxyborohydride. Isolation of the basic fraction gave the desired N,N-disubstituted piperazine which was converted to the corresponding hydrochloride salts using gaseous HCl.

### Receptor binding

All six of the hybrid TFMPP–MDBP regioisomers reported in this manuscript were tested for their serotonin receptor and reuptake pump binding profiles by the University of North Carolina's Psychoactive Drug Screening Project (PDSP). Initially, each isomer was tested in a primary assay at a concentration of 10 umol/L for its ability to displace a standard ligand at each serotonin receptor and transporter subtype. Those compounds which produced greater than 50% binding inhibition in the primary assay were tested further to determine receptor or transporter affinity constants (*K_i_* values in nmol/L) in a secondary binding assay. The *K_i_* (nmol/L) values were obtained from non-linear regression of radioligand competition binding isotherms in this assay and calculated from best-fit inhibitory concentration IC_50_ values using the Cheng–Prusoff equation. The reported values are the average of three (*n* = 3) determinations [16]. Further experimental details regarding these receptor assays are presented in the PDSP Protocol Manual available at the PDSP website.

## Results and discussion

The structures of the six regioisomeric disubstituted 1-phenyl-4-benzyl-piperazines in this study are shown in Figure 1. The N-phenyl group contains the trifluoromethyl-(CF_3_) substituted at the 2-, 3- or 4-position while the N-benzyl aromatic ring contains the methylenedioxy group fused at the 2,3- or 3,4-position. The possible variations/combinations in the position of substitution of these two aromatic ring groups yield the six regioisomeric compounds whose structures are in Figure 1. Compounds 1–3 in Figure 1 contain the three regioisomeric trifluoromethylphenyl substituents and have the methylenedioxy group at the 2,3-position of the N-benzyl aromatic ring. Compounds 4–6 contain identical *ortho, meta* and *para*-trifluoromethylphenyl substituents at one nitrogen and have the 3,4-methylenedioxy-benzyl group at the other nitrogen of the piperazine ring. These six compounds have the same molecular weight as well as identical aromatic ring substituent groups differing only in the relative arrangement of these groups within the 1-phenyl-4-BZP molecular skeleton. Thus, Compounds 2 and 5 represent a combination of the structural features of the monosubstituted piperazine drugs 3-TFMPP and MDBP.

These six compounds were prepared via a one-step N-alkylation of the secondary nitrogen of each of the three isomeric 2-, 3- and 4-TFMPPs. These monosubstituted TFMPPs were reductively aminated individually with 2,3- or 3,4-methylenedioxybenzaldehyde using sodium triacetoxyborohydride as the hydride source to yield the desired disubstituted piperazine derivatives (Compounds 1–6 in Figure 1).

**Figure 2.**
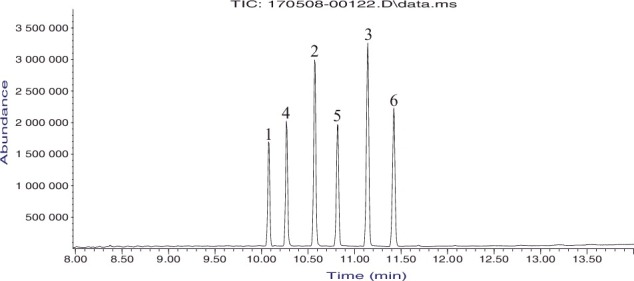
Gas chromatographic separation of the N,N-disubstituted piperazine derivatives on Rxi®−17Sil MS column. The numbers over the peaks correspond to the numbers for the compounds in Figure 1.

The chromatogram in Figure 2 shows the gas chromatographic separation of the six regioisomeric compounds on a capillary column containing the Rxi®−17Sil MS stationary phase. The compounds elute in order of the position of substitution of the trifluoromethyl group as well as the methylenedioxy substitution pattern. The isomer with the trifluoromethyl group at the 2-position of the phenyl ring shows the lowest retention followed by the 3-trifluoromethyl-substituted isomer and finally the 4-trifluoromethyl-substituted isomer has the highest relative retention. Additionally, the 3,4-methylenedioxy group enhances retention compared to the corresponding 2,3-substitution pattern. Furthermore, it is clear from the chromatogram in Figure 2 that the position of the CF_3_ group has the more significant influence on retention. These factors combine to yield a chromatogram with both 2-TFMPP isomers (Compounds 1 and 4) eluting first, followed by the 3-TFMPP isomers (Compounds 2 and 5) and the 4-TFMPP isomers (Compound 3 and 6). The six compounds were well resolved with excellent peak shape on this Rxi®−17Sil MS stationary phase eluting over about a 1.5 min time-window. The identical elution order and similar chromatographic resolution of these six regioisomeric compounds were obtained on columns containing Rtx®−200 and Rtx®−5 stationary phase polymers (chromatograms not shown).

**Figure 3.**
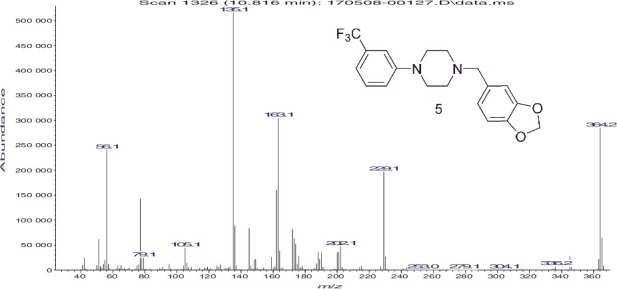
Electron ionization (EI) mass spectrum of one example N,N-disubstituted piperazine.

**Figure 4.**
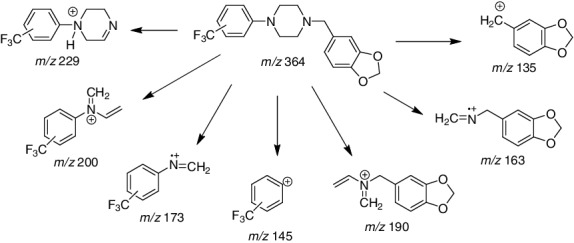
Mass spectral fragmentation pattern of N,N-disubstituted piperazine derivatives under electron ionization (EI) (70 eV) conditions.

The EI-MS for the six regioisomers is shown in Figure 3 and the Supplemental Material. The example spectrum in Figure 3 is representative of the spectra for all six compounds in this study. These compounds each yield a molecular radical cation of significant relative abundance at *m*/*z* = 364 and major fragment ions at *m*/*z* = 135, 163 and 229. The ions at *m*/*z* = 135 and *m*/*z* = 229 represent complimentary fragments via cleavage of the N–C bond between the piperazine ring nitrogen and the benzylic carbon of the methylenedioxybenzyl group. The *m*/*z* = 135 ion represents the methylenedioxybenzyl cation while the loss of the methylenedioxybenzyl radical species with cation formation on the piperazine portion of the structure yields the *m*/*z* = 229 cation fragment. The proposed structures for these fragments as well as all other major ions are shown in Figure 4. The *m*/*z* = 163 major radical cation fragment and the *m*/*z* = 190 cation have been confirmed as containing two and four atoms, respectively, of the piperazine ring as well as the methylenedioxybenzyl group via comparison with the unsubstituted N-benzyl analogue and observation of equivalent mass shifts. The EI-MS spectra for the unsubstituted N-benzyl-2-, 3- and 4-TFMPP derivatives have been described recently in other studies [14].

Equivalent fragmentation processes involving the trifluoromethylphenyl group and two atoms (C–N) or four atoms (C_3_N) of the piperazine ring yield the ion clusters at *m*/*z* = 173 and *m*/*z* = 200, respectively. The low mass ion at *m*/*z* = 56 (C_3_H_6_N)^+^ is characteristic for the piperazine derivatives and previous deuterium labelling studies [17] confirmed the atoms of the piperazine ring as the origin of this fragment. The minor high mass fragments at *m*/*z* = 345 and *m*/*z* = 336 represent the loss of a fluorine radical (M–F)^+^ and ethylene (M–C_2_H_4_)^+^ from the molecular radical cation, respectively.

The mass spectra of these six compounds provide an excellent example of the issue of isomer differentiation and identification in synthetic designer drugs. While some slight differences in relative intensity of the various fragment ions can be observed, these six spectra are essentially identical. There are no unique ions that could be considered characteristic for an individual isomer. These regioisomeric compounds were well resolved via GC as described above; however, chromatography is not a method for confirmation of individual chemical structures. The combined GC–MS results would serve to focus the qualitative analysis on this group of six trifluoromethylphenyl-substituted MDBPs.

The vapour phase IR spectra in Figure 5 provide for individual isomer identification following GC–MS focus on this group of six regioisomeric molecules. These spectra determined in GC–IR experiments provide numerous unique absorption bands for the specific identification of a single isomer within this group. The combination of positioning of both aromatic ring substituent groups yields six unique spectra. The position, shape and relative intensity of numerous absorption bands allow for differentiation among these six molecules having essentially identical mass spectra.
**Figure 5.**
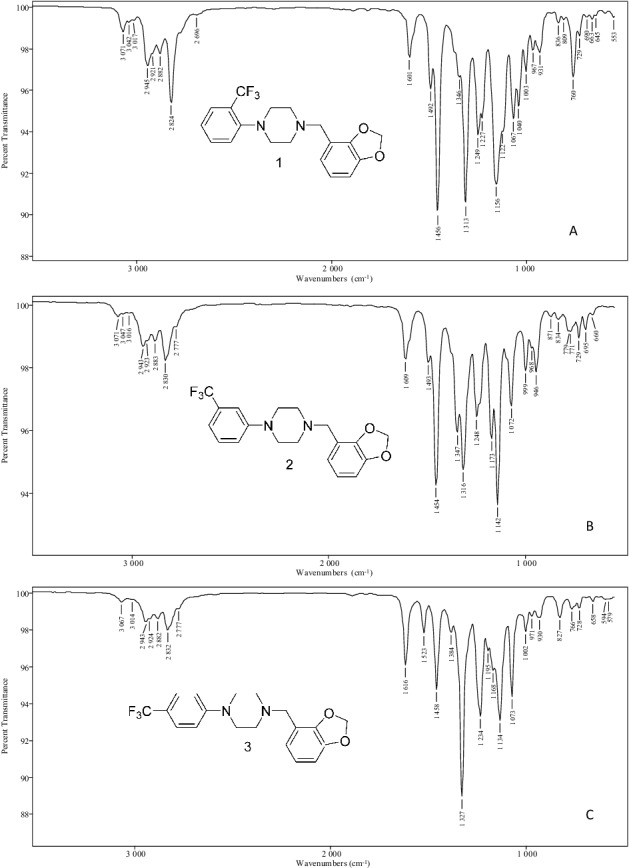
Vapour phase infrared spectra for the N,N-disubstituted piperazine derivatives. Vapour phase infrared spectra for the N-2,3-methylenedioxy-benzyl-2-, 3- and 4-trifluoromethylphenylpiperazines (A–C). Vapour phase infrared spectra for the N-3,4-methylene-dioxybenzyl-2-, 3- and 4-trifluoromethylphenylpiperazines (D–F).

**Figure:**
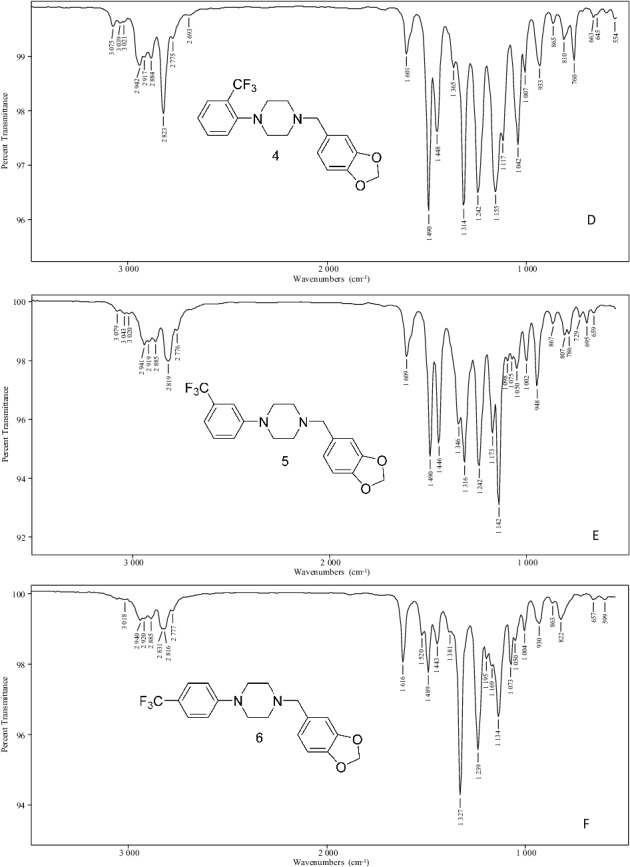

The availability of all six possible substitution patterns allows for a comparison of the influence of regioisomeric structure on the vapour phase IR absorption pattern. A comparison of the spectra for Compounds 1–3 as well as Compounds 4–6 shows the effects of the position of substitution of the trifluoromethyl group on the vapour phase spectrum, while a comparison of the spectra for Compounds 1 and 4, 2 and 5 as well as 3 and 6 shows the effect of the position of the methylenedioxy group. The vapour phase IR spectra for the regioisomeric monosubstituted 2-, 3- and 4-TFMPP analogues as well as the disubstituted N-benzyl-TFMPP analogues have been recently described [13,14].

A number of the major bands observed in Compounds 1–6 are also present in the monosubstituted TFMPP isomers, thus indicating the N-trifluoromethylphenyl group as the major structural feature providing the predominant absorption bands in the vapour phase spectra of Compounds 1–6. The electronic effects of direct nitrogen conjugation to the aromatic ring in this substituted aniline-like moiety yield a number of characteristic vibrational absorbances. The 2-CF_3_ group in both Compounds 1 and 4 produces strong bands in the 1 313 and 1 156 cm^−^^1^ range. Absorbance bands of identical shape and intensity are produced by the monosubstituted 2-TFMPP isomer [13,14]. The 3-CF_3_ group (Compounds 2 and 5) yields unsymmetrical doublets at 1 347/1 316 and 1 173/1 142 cm^−^^1^ consistent with the vapour phase spectrum of the monosubstituted 3-TFMPP. The more symmetrical molecules (Compounds 3 and 6) having the 4-trifluoromethyl aniline-like moiety show a very strong and sharp single absorption band at 1 327 cm^−^^1^ also observed in the vapour phase spectrum of the monosubstituted 4-TFMPP compound [13,14].

The position of the methylenedioxy group in Compounds 1, 2, 4 and 5 is indicated by several peaks including the doublet band in the 1 493–1 446 cm^−^^1^ range. The 2,3-MD isomers (Compounds 1 and 2) show a much stronger absorbance at the lower cm^−^^1^ peak in the 1 456 cm^−^^1^ range with only a shoulder band in the higher 1 492 cm^−^^1^ range. This higher absorbance band at 1 490 cm^−^^1^ is much stronger for the 3,4-MD isomers (Compounds 4 and 5) changing the overall shape of this portion of the spectrum. For Compound 3 (4-TFMPP-2,3-MD), the major absorbance in this range is a single peak at 1 458 cm^−^^1^ while Compound 6, the 3,4-MD-substituted isomer, yields a triplet band with the strongest absorbance occurring at 1 489 cm^−^^1^.

The compounds in this paper are hybrid analogues containing the structural fragments of both 3-TFMPP and 3,4-MDBP drugs of abuse in a single molecule. The parent compound in this series, 3-TFMPP, is believed to produce its pharmacologic effects by interacting with serotonin receptors and serotonin reuptake (SERT) pumps. The 3-TFMPP isomer has been demonstrated to bind with significant affinity to 5-HT_1A_ (*K_i_* = 288 nmol/L), 5-HT_1B_ (*K_i_* = 132 nmol/L), 5-HT_1D_ (*K_i_* = 282 nmol/L), 5-HT_2A_ (*K_i_* = 269 nmol/L) and 5-HT_2C_ (*K_i_* = 62 nmol/L) receptors. This isomer functions as a full agonist at all sites except at the 5-HT_2A_ receptor, where it acts as a weak partial agonist or antagonist. While 3-TFMPP does not show significant affinity for other serotonin receptor subtypes, it does bind to the SERT with an effective concentration (EC_50_) of 121 nmol/L and promotes serotonin release. However, 3-TFMPP has no effects on dopamine and norepinephrine reuptake pumps. At this time, there are no published reports on the receptor pharmacology of 3,4-MDBP.

As the data in Table 1 indicate, in contrast to 3-TFMPP, most of the TFMPP–MDBP isomers did not display significant binding at 5-HT_1_ receptor subtypes (5-HT_1A_, 5-HT_1D_, 5-HT_1E_). Only the 3-TFMPP-3,4-MDBP (Compound 5) isomer displayed affinity comparable to 3-TFMPP at 5-HT_1A_ receptors (*K_i_* = 188 nmol/L). These data indicate that substitution of a methylenedioxybenzyl substituent on the secondary nitrogen atom of 3-TFMPP is detrimental to binding at most 5-HT_1_ receptor subtypes. Most TFMPP–MDBP regioisomers also had significantly reduced affinity at 5-HT_2A_ receptors compared to 3-TFMPP. Only the 3-TFMPP-2,3-MDBP (Compound 2) isomer retained activity comparable to the parent designer drug. All of the TFMPP–MDBP regioisomers displayed significant binding to 5-HT_2B_ receptors, and the 2-TFMPP-2,3-MDBP (Compound 1), 3-TFMPP-2,3-MDBP (Compound 2), 2-TFMPP-3,4-MDBP (Compound 4) and 3-TFMPP-3,4-MDBP (Compound 5) had modest binding to 5-HT_2C_ receptors. None of the TFMPP–MDBP isomers were bound with significant affinity by 5-HT_3_, 5-HT_4_, 5-HT_5A_ and 5-HT_6_ receptor subtypes. However, the 2-TFMPP-2,3-MDBP (Compound 1), 3-TFMPP-2,3-MDBP (Compound 2), 2-TFMPP-3,4-MDBP (Compound 4) and 3-TFMPP-3,4-MDBP (Compound 5) isomers were bound by 5-HT_7_ receptors with the 2-TFMPP-2,3-MDBP isomer having highest affinity.

**Table 1.** Serotonin receptor affinity for the N,N-disubstituted piperazine derivatives.

	Compound number, *K_i_* values (nmol/L)					
Receptor	1	2	3	4	5	6
**5-HT_1A_**	>1 000	864	>1 000	>1 000	188	>1 000
**5-HT_2A_**	>1 000	372	>1 000	>1 000	>1 000	>1 000
**5-HT_2B_**	74.5	203	870	81	116	247
**5-HT_2C_**	868	753	>1 000	924	430	>1 000
**5-HT_7_**	165	747	>1 000	462	516	>1 000
**SERT**	>1 000	>1 000	>1 000	>1 000	>1 000	722

All six isomers had *K_i_* values of >1 000 nmol/L at 5-HT_1B_, 5-HT_1D_, 5-HT_1E_, 5-HT_3_, 5-HT_4_, 5-HT_5A_ and 5-HT_6_; SERT: serotonin reuptake.

Finally, none of these hybrid compounds, except for the 4-TFMPP-3,4-MDBP (Compound 6), had significant affinity for SERT pumps, but even Compound 6 was about six-times less potent than the monosubstituted 3-TFMPP at this transporter. At this time, it is unclear how the differences in serotonin receptor or transporter binding may correlate to any differences in pharmacologic actions between the TFMPP–MDBP isomers and the 3-TFMPP and 3,4-MDBP parent molecules. Testing of these compounds in other receptor systems [16] of the PDSP revealed they had no significant affinity for adrenenergic receptor subtypes (alpha-1, alpha-2, beta-1, beta-2, beta-3), muscarinic receptors (M1–M5), GABA receptors or most dopamine receptor subtypes (D1–D5). All compounds in this series, except the 2-TFMPP-2,3-MDBP (Compound 1) and 3-TFMPP-2,3-MDBP (Compound 2), had affinity values below 200 nmol/L for dopamine D4 receptors. Again, the significance of this affinity relative to pharmacologic activity is unclear at this time.

In summary, a combination of GC–MS and GC–IR data allows for the identification of each of these six regioisomeric disubstituted piperazine derivatives. The EI-MS results allow the analytical focus on this series of six regioisomeric compounds and the vapour phase IR spectra provide specific information to identify each isomer.

## Conclusion

A series of six potential designer N,N-disubstituted piperazines were compared in GC–MS and GC–IR studies. These compounds contain the structural fragments of 1-(3-TFMPP) and 1-(3,4-MDBP) drugs of abuse in a single molecule. These six regioisomeric substances were resolved by capillary GC with elution according to the position of aromatic ring substitution.

The major EI-MS fragment ions at *m*/*z* = 364, 229, 163 and 135 identify these compounds as a member of this set of regioisomeric substances. However, the majority of the EI mass spectral fragment ions occur via processes initiated by one of the two nitrogen atoms of the piperazine ring. The two unsymmetrically substituted piperazine nitrogens each produce fragments containing two and four of the original atoms of the piperazine ring.

The vapour phase IR spectra provide information for confirmation of the position of trifluoromethyl and methylenedioxy substitution on the aromatic rings within the substituted 1-phenyl-4-BZP molecular framework. Thus, the combination of MS and IR data allows for the specific identification of one isomer to the exclusion of the other regioisomers.

The receptor affinity data indicate that addition of a methylenedioxybenzyl substituent on the secondary nitrogen atom of 3-TFMPP is detrimental to binding at most 5-HT_1_ receptor subtypes. However, all of the TFMPP–MDBP regioisomers displayed significant binding to 5-HT_2B_ receptors.

## Supplementary Material

Supp_Files_1445497_TFSR.doc
