# Supplementary material for: Disubstituted piperazine analogues of trifluoromethylphenylpiperazine and methylenedioxybenzylpiperazine: analytical differentiation and serotonin receptor binding studies
Source: Forensic Sci Res. 2018 Apr 5;3(2):161–9. doi: 10.1080/20961790.2018.1445497 (PMC6197089; doi:10.1080/20961790.2018.1445497)
Supplement: Supp_Files_1445497_TFSR.doc [file TFSR_A_1445497_SM4293.doc]

Supplemental Files:

Electron ionization mass spectra of the N,N-disubstituted piperazine derivatives. Compounds 1-4, and 6.
